# A Strigolactone Signal Inhibits Secondary Lateral Root Development in Rice

**DOI:** 10.3389/fpls.2019.01527

**Published:** 2019-11-22

**Authors:** Huwei Sun, Fugui Xu, Xiaoli Guo, Daxia Wu, Xuhong Zhang, Manman Lou, Feifei Luo, Quanzhi Zhao, Guohua Xu, Yali Zhang

**Affiliations:** ^1^Laboratory of Rice Biology in Henan Province, Collaborative Innovation Center of Henan Grain Crops, College of Agronomy, Henan Agricultural University, Zhengzhou, China; ^2^Key Laboratory of Plant Nutrition and Fertilization in Low-Middle Reaches of the Yangtze River, Ministry of Agriculture, Nanjing Agricultural University, Nanjing, China

**Keywords:** auxin, OsPIN2, rice, secondary lateral roots, strigolactones

## Abstract

Strigolactones (SLs) and their derivatives are plant hormones that have recently been identified as regulators of primary lateral root (LR) development. However, whether SLs mediate secondary LR production in rice (*Oryza sativa* L.), and how SLs and auxin interact in this process, remain unclear. In this study, the SL-deficient (*dwarf10*) and SL-insensitive (*dwarf3*) rice mutants and lines overexpressing Os*PIN2* (OE) were used to investigate secondary LR development. The effects of exogenous GR24 (a synthetic SL analogue), 1-naphthylacetic acid (NAA; an exogenous auxin), 1-naphthylphthalamic acid (NPA; a polar auxin transport inhibitor), and abamine (a synthetic SL inhibitor) on rice secondary LR development were investigated. Rice *d* mutants with impaired SL biosynthesis and signaling exhibited increased secondary LR production compared with wild-type (WT) plants. Application of GR24 decreased the numbers of secondary LRs in *dwarf10* (*d10*) plants but not in *dwarf3* (*d3*), plants. These results indicate that SLs negatively regulate rice secondary LR production. Higher expression of *DR5::GUS* and more secondary LR primordia were found in the *d* mutants than in the WT plants. Exogenous NAA application increased expression of *DR5::GUS* in the WT, but had no effect on secondary LR formation. No secondary LRs were recorded in the OE lines, although *DR5::GUS* levels were higher than in the WT plants. However, on application of NPA, the numbers of secondary LRs were reduced in *d10* and *d3* mutants. Application of NAA increased the number of secondary LRs in the *d* mutants. GR24 eliminated the effect of NAA on secondary LR development in the *d10*, but not in the *d3*, mutants. These results demonstrate the importance of auxin in secondary LR formation, and that this process is inhibited by SLs *via* the *D3* response pathway, but the interaction between auxin and SLs is complex.

## Introduction

Plants have successfully colonized the terrestrial environment *via* the evolution of multicellular organs that absorb the nutrients and water required for their growth and development (Pires and Dolan, 2012). The root system is the main organ by which plants obtain nutrients and water from soil (Péret et al., 2009; Sun et al., 2018a, 2018b; Huang et al., 2019). Therefore, diversity and plasticity in root architecture may contribute to the survival strategies of plants.

Root systems consist of embryonic roots derived from the embryo and post-embryonic roots derived from existing roots or non-root tissues (Atkinson et al., 2014). Post-embryonic roots arising from existing roots are lateral roots (LRs), and roots arising from non-root tissues are adventitious roots (ARs) (Atkinson et al., 2014). LRs develop from founder cells in the pericycle, the outermost layer of the vascular cylinder (stele) of a root (De Smet et al., 2006). In contrast to the taproot system, the majority of monocot roots form a fibrous root system that is characterized by the formation of many seminal roots (SRs) and ARs. In monocots, the LRs develop from ARs and SRs (Osmont et al., 2007; Bellini et al., 2014).

Several lines of study have suggested that LR formation is regulated by genetic factors (Lavenus et al., 2015; Murphy et al., 2016; Fernández-Marcos et al., 2017). In addition to genetic factors, LR growth and development are also regulated by plant hormones, such as auxin. Previous studies have shown that auxin plays a key role in LR formation and growth in plants (Guseman et al., 2015; Xuan et al., 2016; Tang et al., 2017). Auxin is synthesized mainly in aboveground tissue, such as shoot apices, by *YUCCA* family genes (Zhao, 2012) and redistributed by auxin-influx carriers, such as AUX1/LAX family proteins, and auxin-efflux carriers, including ABCB/PGP and PIN family proteins in several plant species (Friml, 2003; Blakeslee et al., 2005; Wang et al., 2009; Zazimalova et al., 2010; Péret et al., 2012). The polar transport of auxin is very important for LR development in plants (Swarup et al., 2005; De Smet et al., 2007; Inahashia et al., 2018). For example, the roots of the *aux1* mutant bend constitutively in one direction, forming root coils with LRs distributed predominantly on the convex side of the curve, which differs markedly from the wavy pattern seen in the roots of *Arabidopsis* (Swarup et al., 2005; De Smet et al., 2007). The mutants of *pin2*, *pin3*, and *pin7* have an altered branching pattern, with closely grouped lateral root primordia (LRP)/LRs or fewer LRP/LRs, in *Arabidopsis* (Laskowski et al., 2008). Moreover, *AtPIN3* is part of an auxin reflux pathway that is transiently established during the early phases of LR formation (Marhavy et al., 2013). *OsPIN2*-altered auxin flow in the root tip region is responsible for LR growth and formation patterns in rice (Inahashia et al., 2018).

Besides auxin, newly identified phytohormones named strigolactones (SLs) are involved in the growth and formation of LRs in several plant species (Kapulnik et al., 2011; Ruyter-Spira et al., 2011; Mayzlish-Gati et al., 2012; Rasmussen et al., 2012; Sun et al., 2014; De Cuyper et al., 2015). Compared with WT plants, a SL-synthesis mutant (*more axillary growth4*) and a SL-signaling mutant (*more axillary growth2*) were found to have greater LR densities in *Arabidopsis* (Kapulnik et al., 2011; Kohlen et al., 2011; Ruyter-Spira et al., 2011). However, LR density did not differ between WT plants and *d* mutants in rice (Sun et al., 2014). In *Arabidopsis* and rice, application of GR24 (a SL analogue) decreased the LR density in WT plants and SL-synthesis mutants (*more axillary growth4/d10*), but not in SL-signaling mutants (*more axillary growth2/d3*) (Ruyter-Spira et al., 2011; Sun et al., 2014).

The interactions between SLs and auxin in the regulation of LR growth are closely linked (Ruyter-Spira et al., 2011; Sun et al., 2014). In *Arabidopsis* and rice, higher auxin levels in roots were recorded in SL-synthesis mutants than in WT plants. Application of GR24 to the roots of WT and SL-synthesis mutants inhibited LR formation by reducing auxin transport (Ruyter-Spira et al., 2011). PIN proteins are the major auxin efflux carriers in plants (Friml, 2003; Wisniewska et al., 2006). Application of GR24 decreased PIN1, PIN3, and PIN7 protein levels in the primary root tips of *Arabidopsis*. However, PIN levels were not affected when similar levels of GR24 were applied in the presence of exogenous auxin (Ruyter-Spira et al., 2011). The expression of most *PIN* family genes in roots was downregulated by application of GR24 in rice (Sun et al., 2014). These results indicate that SLs inhibit LR formation, perhaps by reducing the levels of PIN proteins.

Rice is an ideal model for the study of plant root growth because of its small genome and the availability of its complete genome sequence and well-characterized mutants (Feng et al., 2002; Sasaki et al., 2002). Relative to primary LR development, the formation of secondary LRs in rice has not been characterized in detail. We found that secondary LR formation was induced in *d* mutants and that exogenous GR24 inhibited secondary LR formation in *d10* plants, but not in *d3* plants. NPA treatment reduced the number of secondary LRs in the *d* mutants. However, application of NAA increased the number of secondary LRs in the *d* mutants, but not in WT plants. The effect of NAA on secondary LR development was eliminated by supplying GR24 to the *d10* plants, but this was not the case in the *d3* plants. These results demonstrate that auxin induced rice secondary LR formation in the absence of SLs.

## Materials and Methods

### Plant Materials

The *d3-1* and *d10-1* mutants (Shiokari ecotype) (Sun et al., 2014), and lines overexpressing *OsPIN2* (OE1 and OE2) (Nipponbare ecotype), were used in this study.

### Plant Growth

Rice seedlings were grown at day/night temperatures of 30/18°C under natural light in a greenhouse. Seven-day-old seedlings of uniform size and vigour were transplanted into holes in lids placed over the tops of pots (four holes per lid and three seedlings per hole). Nutrient solutions ranging from one-quarter strength to full strength were applied for 1 week, followed by application of full-strength nutrient solution for 2 weeks. The chemical composition of the International Rice Research Institute (IRRI) nutrient solution is (mM): 1.25 (NH_4_)_2_SO_4_, 0.3 KH_2_PO_4_, 0.35 K_2_SO_4_, 1.0 CaCl_2_, 1.0 MgSO_4_·7H_2_O, 0.5 Na_2_SiO_3_; and (µM) 9.0 MnCl_2_, 0.39 (NH_4_)_6_Mo_7_O_24_, 20.0 H_3_BO_3_, 0.77 ZnSO_4_, and 0.32 CuSO_4_ (pH 5.5).

The treatments applied were as follows: 10 nM 1-naphthylacetic acid (NAA), 2.5 µM GR24 (a SL analogue), 100 µM abamine (a SL-synthesis inhibitor) (Sun et al., 2014; Sun et al., 2015; Sun et al., 2016), and localized application of NPA (a polar auxin transport inhibitor). The latter was by dispensing diluted agar containing 20 µM NPA directly from a pipette across the shoot base (Chen et al., 2012). All experiments included three independent biological replicates.

### Measurement of Secondary Lateral Root and Primordia Numbers

As reported previously, SRs were significantly longer than ARs under our experimental conditions. Our preliminary experiment showed similar primary LR and secondary LR responses in SRs and ARs (Sun et al., 2014). Therefore, SRs were chosen as representative organs to study the mechanism of secondary LR formation. Primary LR density and the numbers of secondary LRs/primordia SR were analyzed in detail. SR length was measured with a ruler and LRs/secondary LRs were counted by eye. Primary LR density was calculated as LR number divided by SR length. All experiments included three independent biological replicates.

In this study, the stages of secondary LR development followed Malamy and Benfey (1997), with stages I–XII grouped here as unemerged primordia. The primordia of the secondary LRs were classified as unemerged and emerged. An emerged LRP longer than 0.5 mm (visible to the naked eye) was considered a LR, and was referred to as being activated (Song et al., 2013). To visualize the development of secondary LRs, we exploited *pDR5::GUS* transgenic rice plants. After the roots were stained in GUS buffer solution, the secondary LR primordia were easy to count. The experiments included three independent biological replicates.

### *pDR5*::GUS Construct

To examine the distribution of indole-3-acetic acid (IAA) in rice plants, the *pDR5::GUS* construct was transformed into the WT plants a, *d* mutants, and lines overexpressing *OsPIN2* using *Agrobacterium tumefaciens* (strain EHA105) (Sun et al., 2014). The samples used for IAA analysis were also used for histochemical GUS staining. The stained tissues were photographed using an Olympus SZX2-ILLK stereomicroscope with a color CCD camera (Olympus).

GUS activity was examined according to Jia et al. (2011). Samples were homogenized in GUS extraction buffer (50 mM NaPO_4_ (pH 7.0), 10 mM 2-mercaptoethanol, 10 mM Na_2_-EDTA, 0.1% sodium dodecyl sulfate, 0.1% Triton X-100). After centrifugation, 20 µl of the supernatant was mixed with 180 µl of an assay buffer containing 1 mM 4-methylumbelliferyl-β--glucuronide. After incubation at 37°C for 1 h, the reaction was stopped by adding 1,800 µl 0.2 M Na_2_CO_3_. Fluorometer values were compared with those of a 4-methylumbelliferone dilution series. Protein content was determined with a Bio-Rad protein assay kit (Bio-Rad Laboratories, Shanghai, China) using bovine serum albumin as the standard. All experiments included three independent biological replicates.

### Strigolactone Measurement

After 3 weeks growth, root exudates (approximately 500 ml) of the rice plants were collected at 24-h intervals, as described previously (Yoneyama et al., 2012; Xie et al., 2013). Root exudates adsorbed on charcoal were eluted with acetone. After evaporation of the acetone *in vacuo*, the residue was dissolved in 50 ml water and extracted three times with 50 ml ethyl acetate. The ethyl acetate extracts were combined, washed with 0.2 M K_2_HPO_4_ (pH 8.3), dried over anhydrous MgSO_4_, and concentrated *in vacuo*. These crude extracts were stored in sealed glass vials at 4°C until use.

The 2’-*epi*-5-deoxystrigol concentrations in the root exudates were determined by liquid chromatography–mass spectrometry/mass spectrometry, as described previously (Xie et al., 2013). Data were acquired and analyzed using MassLynx software (ver. 4.1; Waters, Milford, MA). The experiments included three independent biological replicates.

### Quantitative Reverse Transcription-Polymerase Chain Reaction

Total RNA was isolated from the roots of 7-day-old rice plants. The RNA extraction, reverse transcription, and quantitative reverse transcription-polymerase chain reaction (qRT-PCR) methods were as described by Jia et al. (2011). The experiments included three independent biological replicates.

### Data Analysis

Data from the experiments were pooled to calculate the means and standard errors (SEs) and subjected to one-way analysis of variance (ANOVA), followed by an LSD test at *P < 0.05* to determine the statistical significance of differences between treatments. All statistical evaluations were conducted using SPSS (version 11.0) statistical software (SPSS Inc., Chicago, IL, USA).

## Results

### Tiller and Secondary Lateral Root Production Were Induced in the Rice *dwarf3* and *dwarf10* Mutants

**Figure 1:**
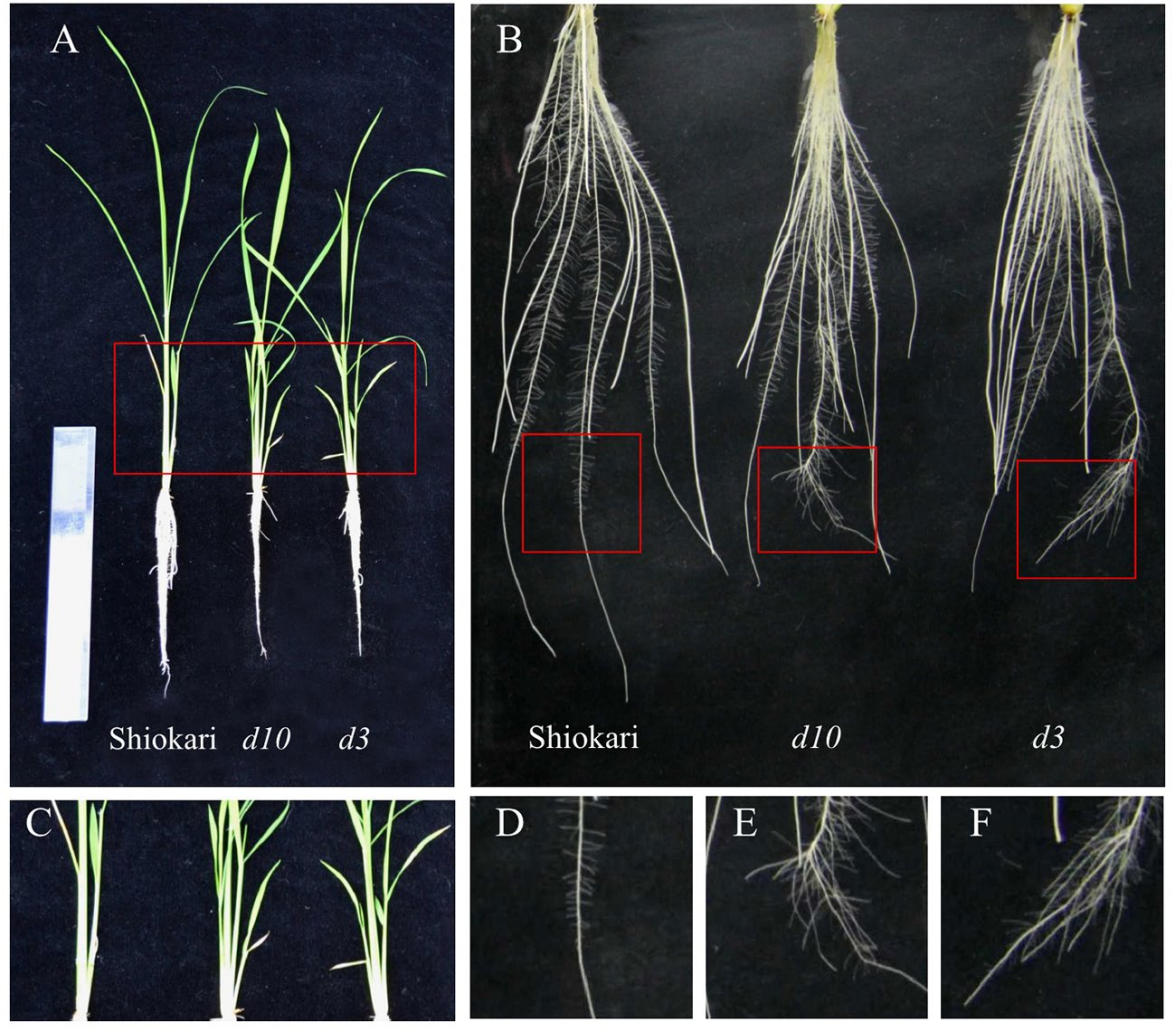
The morphology of tiller and roots in wild-type (WT, Shiokari), strigolactone-synthesis (*d10*), and strigolactone-signaling (*d3*) mutants. Seedlings were grown in a hydroponic media for 21 days. **(A)** The morphology of the rice plants. **(B)** The morphology of roots. **(C)** Tiller. **(D**–**F)** Secondary lateral root (LR). All experiments included three independent biological replicates.

As reported by Ishikawa et al. (2005), compared with wild-type (WT) plants, tiller numbers were increased in *d10* (SL-synthesis mutant) and *d3* (signaling mutant) plants (**Figures 1A, C**). Secondary LR formation was significantly induced in the *d* mutants relative to the WT plants (**Figures 1B, D–F**). These results imply that SLs induce branching of both shoots and roots.

### Exogenous Application of GR24 Inhibited Secondary Lateral Root Formation in the *dwarf10* Plants, But Not in the *dwarf3* of Rice

**Figure 2:**
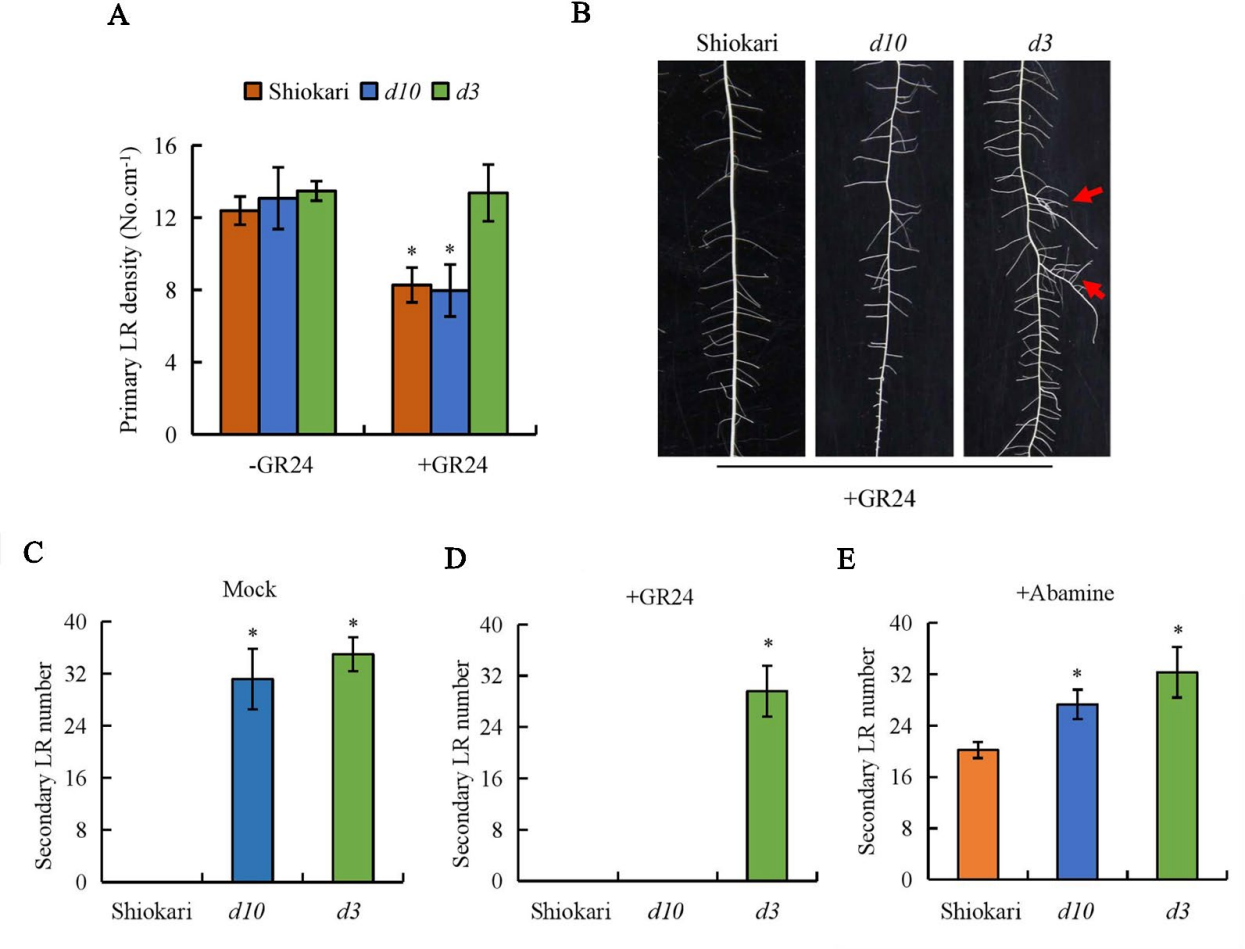
The primary lateral root (LR) density and secondary LR number in wild-type (WT, Shiokari), strigolactone-synthesis (*d10*), and strigolactone-signaling (*d3*) mutants. Seedlings were grown in a hydroponic media with or without GR24 and Abamine for 21 days. **(A)** Primary LR density. **(B)** The morphology of secondary LR number. **(C**–**E)** Secondary LR number. Data are means ± SE. *P < 0.05 comparing the WT and other rice plants. The red arrow indicates the secondary LR. All experiments included three independent biological replicates.

As in a previous study, endogenous 2’-*epi*-5-deoxystrigol was detected in WT and *d3* mutant plants, but not in *d10* plants (Umehara et al., 2008). Primary LR density of SRs did not differ between the WT plants and the *d* mutants (**Figure 2A**). Application of GR24 decreased the primary LR density in the WT and *d10* mutant plants, but not in the *d3* plants (**Figure 2B**). These results are consistent with those reported by Sun et al. (2014). To determine whether SLs regulate the formation of secondary LRs in rice, GR24 was applied exogenously to WT plants and the two *d* mutants (**Figures 2A, B**). Application of GR24 had no effect on the development of secondary LRs in the WT, but inhibited secondary LR formation in the *d10* mutants to the same level of the WT plants. However, the numbers of secondary LRs in the *d3* mutants were not affected by GR24 application (**Figures 2C, D**). Treatment with abamine had no effect on the development of secondary LRs in the *d* mutants, but induced secondary LR formation in the WT plants (**Figure 2E**). These results indicate that SLs inhibit secondary LR formation and the involvement of SL signaling (*D3* gene) in the SL regulatory pathway for secondary LR formation.

### Higher Auxin Levels in the Rice Roots Were Not the Only Reason for Secondary LR Formation

**Figure 3:**
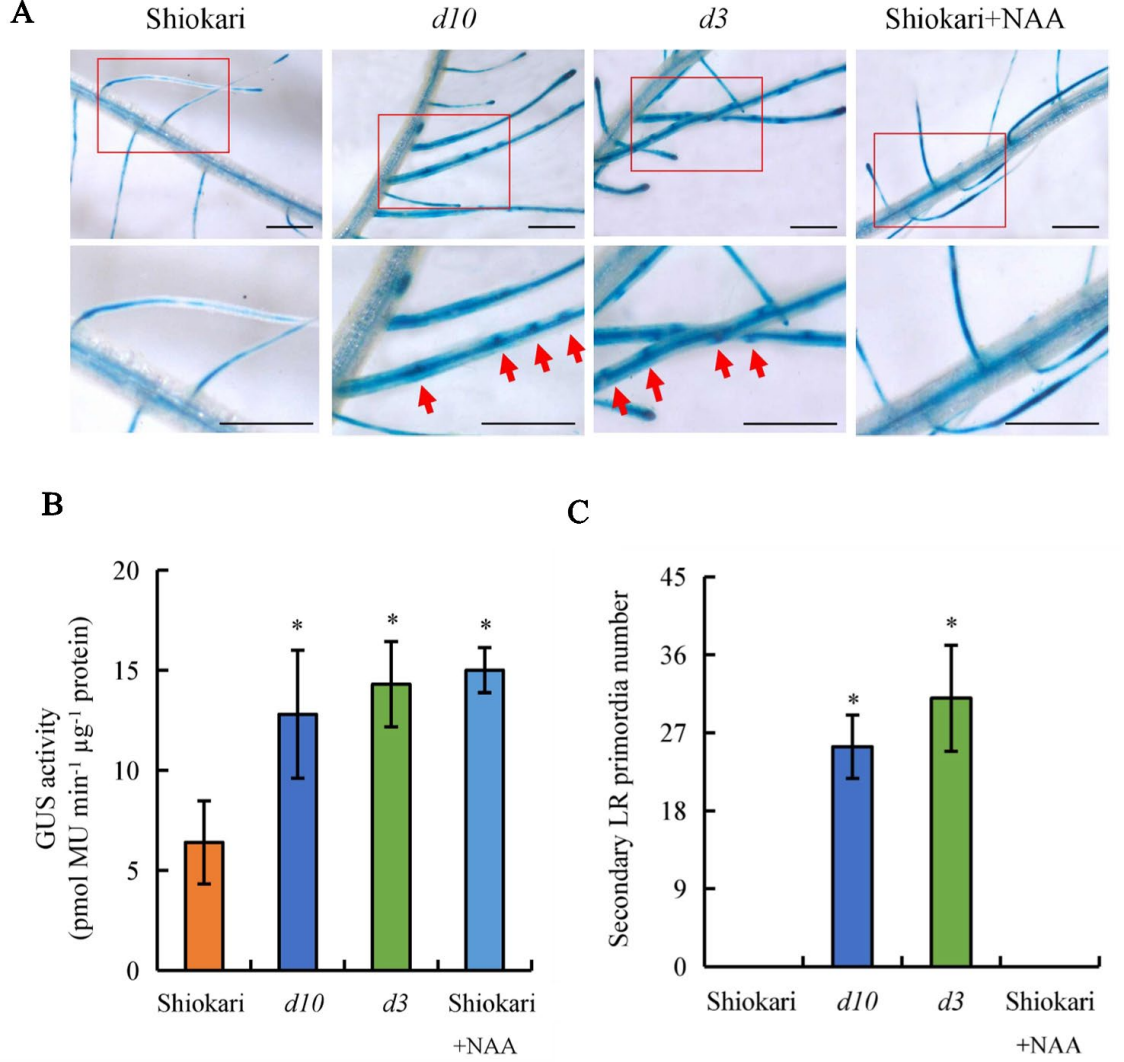
*DR5::GUS* activity and secondary lateral root (LR) primordia number in rice plants. Seedlings were grown in a hydroponic media with or without 1-naphthylacetic acid for 21 days. **(A**, **B)** *DR5::GUS* activity in LR region. **(C)** Secondary LR primordia number. Bar = 1 mm. Data are means ± SE. *P < 0.05 comparing the WT and other rice plants. The red arrow indicates the secondary LR primordia. All experiments included three independent biological replicates.

In a previous study, endogenous IAA levels were higher in the roots of *d10* and *d3* mutants relative to WT plants (Sun et al., 2014). To assess whether higher auxin levels induce secondary LR formation in *d* mutants, the secondary LR primordia in the roots of rice plants were analyzed on application of exogenous NAA. A specific reporter was used that contains seven repeats of a highly active synthetic auxin response element, and changes in auxin levels *in vivo* were monitored *via* the expression of *DR5::GUS* (Ulmasov et al., 1997). Expression of *DR5::GUS* was subsequently examined in the WT plants and in the *d10* and *d3* mutants. GUS activity was higher in the roots of the *d* mutants than in the WT plants (**Figures 3A, B**), consistent with Sun et al. (2014). However, the numbers of secondary LR primordia were significantly higher in the *d* mutants, but not in the WT plants (**Figures 3A, C**). These results imply that higher auxin levels in roots increase secondary LR primordia production. Application of NAA to the WT plants increased expression of *DR5::GUS* in roots to levels similar to those in the roots of the *d* mutants (**Figure 3C**). However, the higher *DR5::GUS* levels did not induce secondary LR primordia formation in the WT plants. These results suggest that higher auxin levels in the roots of *d* mutants were not the only reason for secondary LR primordia formation.

### Overexpression of *OsPIN2* Increased Auxin Levels in Roots But Did Not Induce Secondary Lateral Root Formation

**Figure 4:**
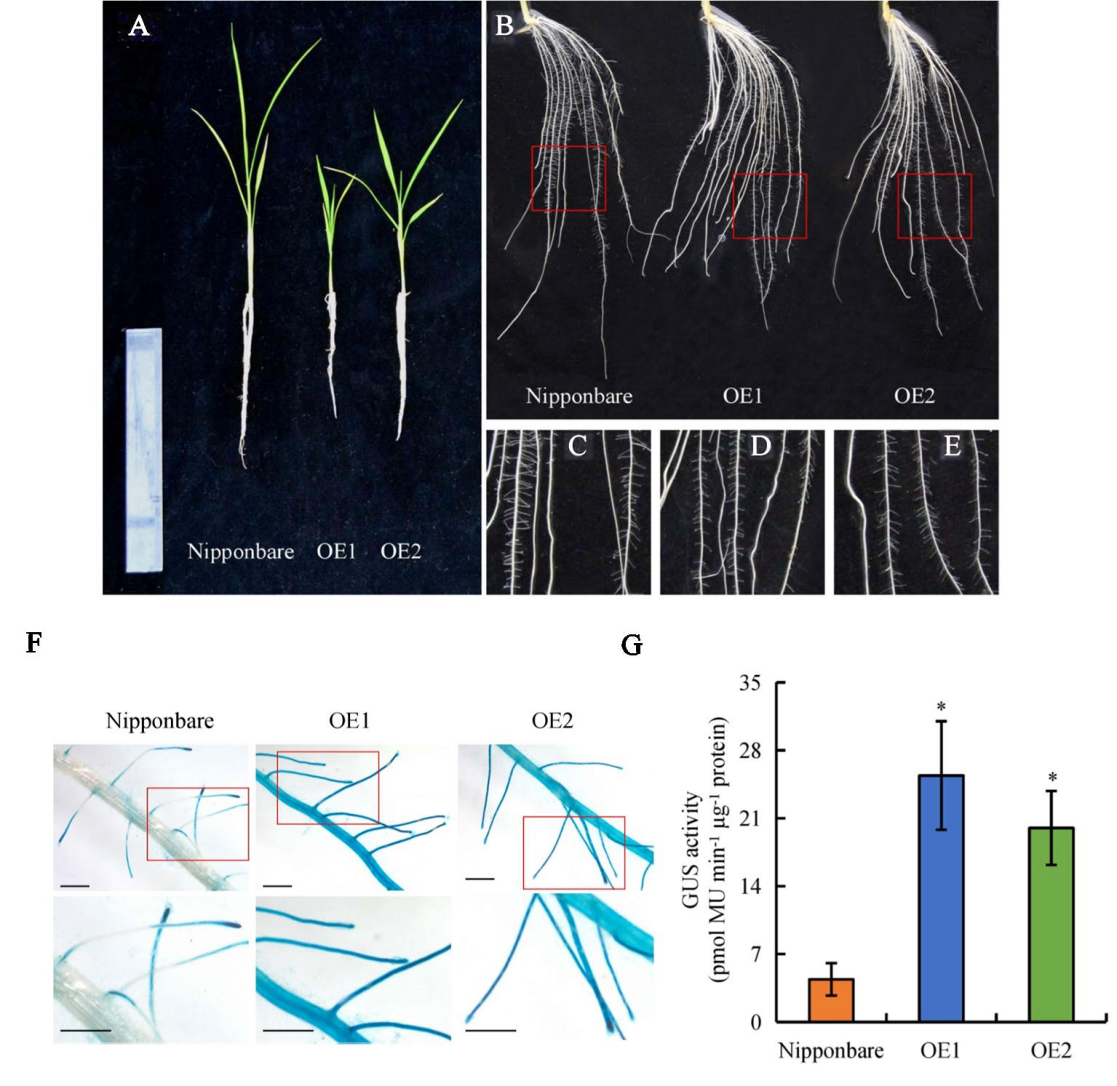
The morphology and *DR5::GUS* activity in wild-type (WT, Nipponbare) and overexpression of *OsPIN2* lines (OE1/OE2). Seedlings were grown in a hydroponic media for 21 days. **(A)** The morphology of the rice plants. **(B**–**E)** The morphology of roots. **(F**, **G)** *DR5::GUS* activity in lateral root region. Bar = 1 mm. Data are means ± SE. *P < 0.05 comparing the WT and other rice plants. All experiments included three independent biological replicates.

**Figure 5:**
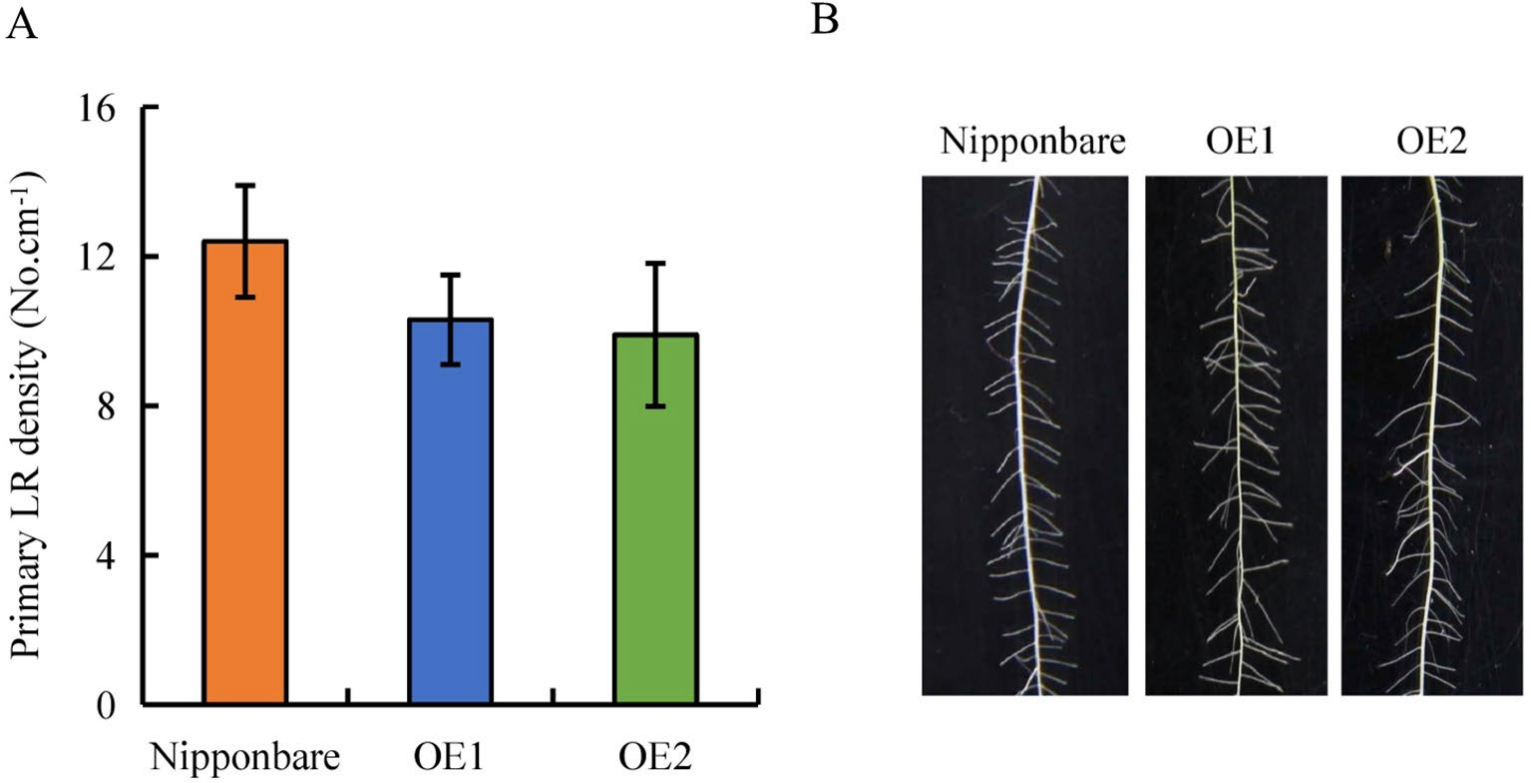
Lateral root (LR) region in wild-type (WT, Nipponbare) and overexpression of *OsPIN2* lines (OE1/OE2). Seedlings were grown in a hydroponic media for 21 days. **(A)** Primary LR density. **(B)** The morphology of LRs region. Data are means ± SE. All experiments included three independent biological replicates.

Expression of *OsPIN2* was analyzed in the WT plants and in the *d* mutants. Compared with the WT plants, levels of *OsPIN2* were up-regulated in the *d* mutants (**Supplementary Figure 1**). To determine further whether secondary LRs were induced by auxin, lines overexpressing *OsPIN2* were used in this study. As reported by Chen et al. (2012), compared with the WT, plant height was significantly reduced in lines overexpressing *OsPIN2* (OE) (**Figure 4A**). The endogenous IAA content of roots is higher in OE lines than in WT plants (Chen et al., 2012). The secondary LR primordia in the roots of the OE lines were analyzed and expression of *DR5::GUS* in the rice plants was subsequently examined. GUS activity was higher in the roots of the OE lines than in those of the WT plants (**Figures 4F, G**), consistent with Chen et al. (2012). However, no secondary LRs or LR primordia were found in the OE lines (**Figures 4B-F** and **5B**). In addition, the primary LR density and numbers of secondary LRs did not differ between the WT and OE lines (**Figures 5A, B**). These results further imply that the higher auxin levels in the OE lines did not induce the development of secondary LRs.

### Auxin Induced the Development of Secondary Lateral Roots in the Absence of Strigolactones

**Figure 6:**
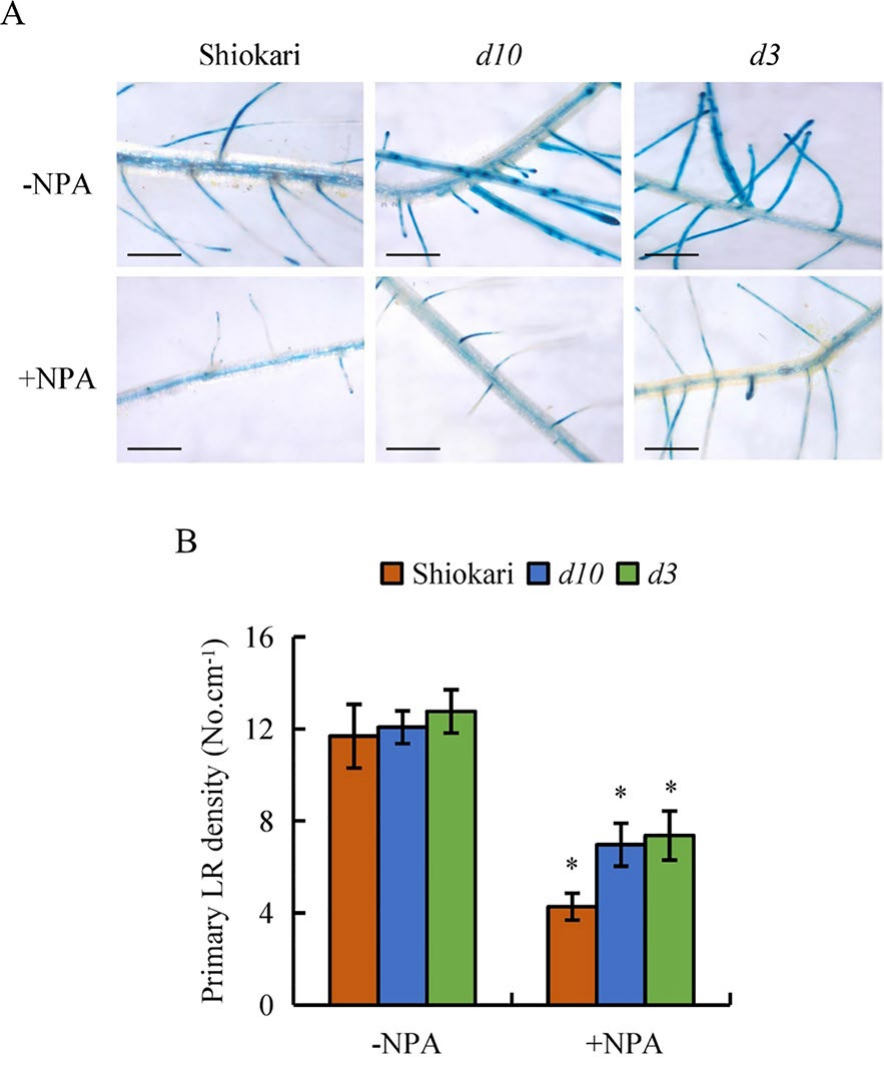
*DR5::GUS* activity and lateral root (LR) region in wild-type (WT, Shiokari) and *d* mutants. Seedlings were grown in a hydroponic media with or without NPA for 21 days. **(A)**, *DR5::GUS* activity in LR region. **(B)** Primary LR density. Bar = 1 mm. Data are means ± SE. *P < 0.05 comparing the WT and other rice plants. All experiments included three independent biological replicates.

**Figure 7:**
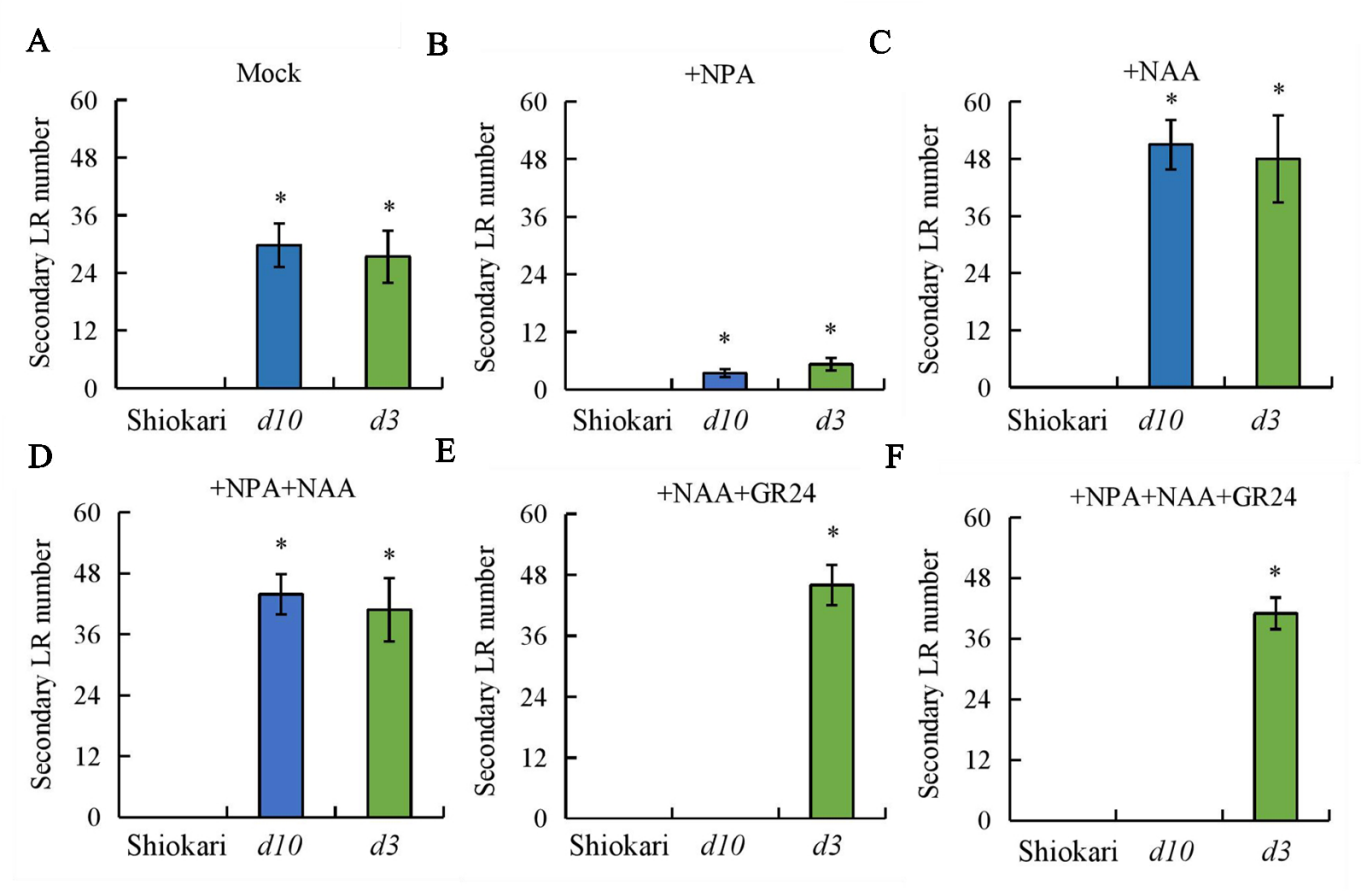
The secondary lateral root (LR) number in wild-type (WT, Shiokari) and *d* mutants. Seedlings were grown in a hydroponic media in addition to NPA, NAA, NPA+NAA, NAA+GR24, and NPA+NAA+GR24 for 21 days. **(A**–**F)** Secondary lateral root number. Data are means ± SE. *P < 0.05 comparing the WT and other rice plants. All experiments included three independent biological replicates.

To analyze further the interaction between SLs and auxin in the regulation of secondary LR development, the numbers of secondary LRs in the WT plants and the *d* mutants were recorded on application of NAA, NPA, NPA+NAA, NAA+GR24, and NPA+NAA+GR24. In comparison with mock treatment, application of NPA significantly decreased both *DR5::GUS* levels in the primary LR region and primary LR density in the WT plants and in the *d* mutants (**Figures 6A, B**). The numbers of secondary LRs were reduced in the *d10* and *d3* mutants under NPA treatment relative to the mock condition (**Figure 7B**). The numbers of secondary LRs were increased in the *d* mutants, but not in the WT plants, on NAA supply (**Figure 7C**). Application of NAA restored the effect of NPA on the numbers of secondary LRs to levels similar to those induced by NAA treatment alone in the *d* mutants (**Figure 7D**), and supply of GR24 eliminated the effect of NAA on secondary LR development (**Figure 7E**). Treatment of roots with GR24 under the NPA plus NAA condition further inhibited secondary LR formation in the *d10* plants, but not in the *d3*, mutants (**Figure 7F**). These results imply that auxin induces secondary LR formation in the absence of SLs.

## Discussion

Development of optimal root morphology, including formation of LRs, is crucial for absorbance of nutrients and water and successful growth of transplants. In addition to providing anchorage, LRs contribute to water-use efficiency and facilitate extraction of micro- and macronutrients from soil (Casimiro et al., 2001; Péret et al., 2009). Most studies of LRs in plants have focused on primary LRs; the mechanisms of secondary LR formation remain largely unexplored. This study provides evidence of the regulatory roles of auxin and SLs in rice secondary LR development.

The SL pathway is involved in primary LR growth and development. In tomato (*Solanum lycocarpum*) and *Arabidopsis*, the density of primary LRs was increased in SL mutants, implying that SLs inhibit LR formation (Koltai et al., 2010; Kapulnik et al., 2011; Ruyter-Spira et al., 2011). Application of GR24 reduced primary LR formation by suppressing the outgrowth of primary LRs in *Arabidopsis* and pea (Kapulnik et al., 2011; Ruyter-Spira et al., 2011; Rasmussen et al., 2012). The density of primary LRs was affected by GR24 in WT seedlings and SL-synthesis mutants, but not in SL-signaling mutants, implying that the effect of SLs on primary LR density is mediated *via* the *MAX2* gene (Kapulnik et al., 2011; Koltai, 2011; Ruyter-Spira et al., 2011). In contrast to findings in upland plants, primary LR density did not differ between WT plants and *d* mutants in rice (Arite et al., 2012; Sun et al., 2014). Application of GR24 reduced primary LR density, but the extent of the decrease did not change with increasing GR24 concentrations (Sun et al., 2014). The primary LR densities in the *d* mutants in the present study were similar to those reported by Sun et al. (2014). Correspondingly, the numbers of secondary LRs increased significantly in the *d* mutants relative to the WT plants. Application of GR24 reduced the numbers of secondary LRs in the *d10* mutants, but not in the *d3* mutants (**Figures 2C, D**). Treatment with abamine induced secondary LR formation in the WT plants (**Figure 2E**). These results indicate that SLs inhibit secondary LR formation and demonstrate the involvement of the *D3* gene in the SL regulatory pathway for secondary LR formation.

Accumulating evidence indicates that auxin regulates LR formation in plants (Goh et al., 2012; Xuan et al., 2016). Polar auxin transport is essential for LR formation, and an auxin-transport-independent pathway is involved in changes in LR formation in plants (Swarup et al., 2005; De Smet et al., 2007; Okumura et al., 2013; Inahashia et al., 2018). However, the mechanisms by which auxin regulates secondary LR formation are poorly understood. In this study, *DR5::GUS* levels were higher in the roots of the *d* mutants than in the WT plants (**Figures 3A, B**), consistent with a report by Sun et al. (2014). These results imply that the higher auxin levels in the roots of *d* mutants are not the reason for secondary LR formation. In addition, lines overexpressing *PIN2* showed increased auxin transport from shoots to roots in rice (Chen et al., 2012). Similar to the *d* mutants, higher *DR5::GUS* levels were found in roots in the OE lines relative to the WT plants (**Figures 4F, G**). However, no secondary LRs were induced in the OE lines (**Figure 5B**). These results further imply that higher auxin levels in roots may not be the reason for secondary LR formation in rice.

It has been suggested that SLs modulate auxin transport, thereby regulating primary LR growth (Ruyter-Spira et al., 2011; Sun et al., 2014). Polar auxin transport is mediated primarily by *PIN* genes. In *Arabidopsis*, Ruyter-Spira et al. (2011) suggested that SLs modulate local auxin levels and that the net result of SL action is dependent on the auxin status of the plant. Application of GR24 inhibited primary LR formation by decreasing auxin transport in roots, with the involvement of PIN protein (Ruyter-Spira et al., 2011; Sun et al., 2014). Experiments examining [^3^H]IAA transport and *DR5::GUS* activity confirmed that application of GR24 markedly reduced auxin transport, indicating that *PINs* are involved in the auxin transport from the shoots to the roots that is downregulated by SLs in rice (Sun et al., 2014; Sun et al., 2018b). In this study, similar SL levels were recorded in WT plants and in lines overexpressing *OsPIN2* (**Supplementary Figure 2**). Although higher auxin levels were found in OE lines than in WT plants (Chen et al., 2012; **Figures 4F, G**), no secondary LRs were induced in the OE lines (**Figure 5**). Application of NPA significantly decreased *DR5::GUS* levels in the primary LR region and the density of primary LRs in the WT plants and in the *d* mutants (**Figures 6A, B**). However, the numbers of secondary LRs were reduced in the *d* mutants under NPA treatment (**Figures 7A, B**). Treatment with NAA restored the effect of NPA on the numbers of secondary LRs in the *d* mutants (**Figure 7C**). These results imply that auxin is involved in the development of secondary LRs. The effect of NAA on secondary LR development was eliminated in the *d10* mutants, but not in the *d3* mutants, by application of GR24 (**Figure 7E**). And a model for these signaling pathways is shown in **Supplementary Figure 3**. These results further demonstrate that secondary LR formation is inhibited by SLs *via* the *D3* response pathway, and the importance of auxin for secondary LR formation.

## Data Availability Statement

All datasets generated for this study are included in the article/**Supplementary Material**.

## Author Contributions

HS performed experiments. FX, XG, DW, XZ, ML and FL assisted the experiment. QZ analyzed data. HS, GX, and YZ designed the experiment. HS wrote the paper.

## Funding

This work was funded by the National Nature Science Foundation of China (Grant No 31601821 and 31672225), the 111 Project (12009), the Innovative Research Team Development Plan of the Ministry of Education of China, the PAPD project of the Jiangsu Higher Education Institutions, and the Jiangsu Overseas Research & Training Program for University Prominent Young & Middle-Aged Teachers and Presidents. Modern Agricultural Industry Technology System Projects of Henan Province (S2012-04-02). The English in this document has been checked by at least two professional editors, both native speakers of English. For a certificate, http://www.textcheck.com/certificate/oMpqOI.

## Conflict of Interest

The authors declare that the research was conducted in the absence of any commercial or financial relationships that could be construed as a potential conflict of interest.
