# Supplementary material for: A Strigolactone Signal Inhibits Secondary Lateral Root Development in Rice
Source: Front Plant Sci. 2019 Nov 22;10:1527. doi: 10.3389/fpls.2019.01527 (PMC6882917; doi:10.3389/fpls.2019.01527)
Supplement: Supplementary file 1 [file DataSheet_1.pdf]

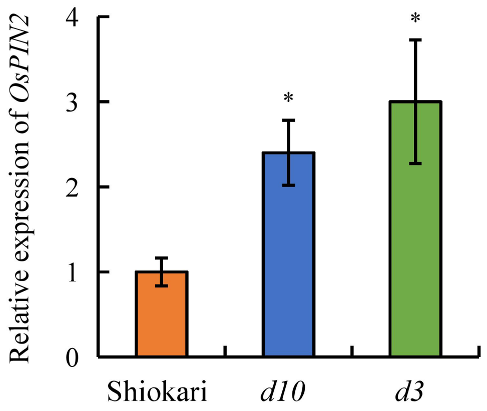

Supplementary FIGURE 1 qRT-PCR analysis of *OsPIN2* gene in the roots of wild-type (WT) and strigolactone-deficient (*d10*) and strigolactone-insensitive (*d3*) mutant rice plants. Seedlings were grown in a hydroponic media for 21 days. Data are means ± SE. *, $P < 0.05$ comparing the WT and other rice plants.

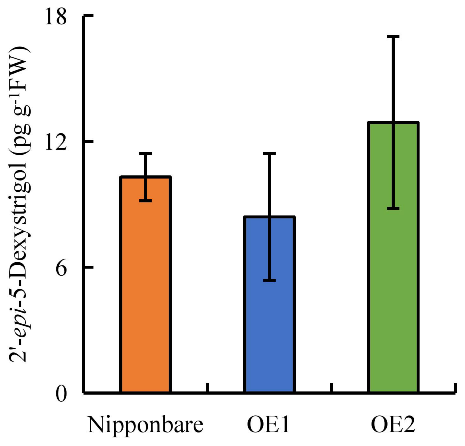

Supplementary FIGURE 2 Levels of 2'-*epi*-5-Deoxystrigol in wild-type (WT, Nipponbare) and overexpression of *OsPIN2* lines (OE). Seedlings were grown in a hydroponic media for 21 days. Data are means ± SE. *, $P < 0.05$ comparing the WT and other rice plants.

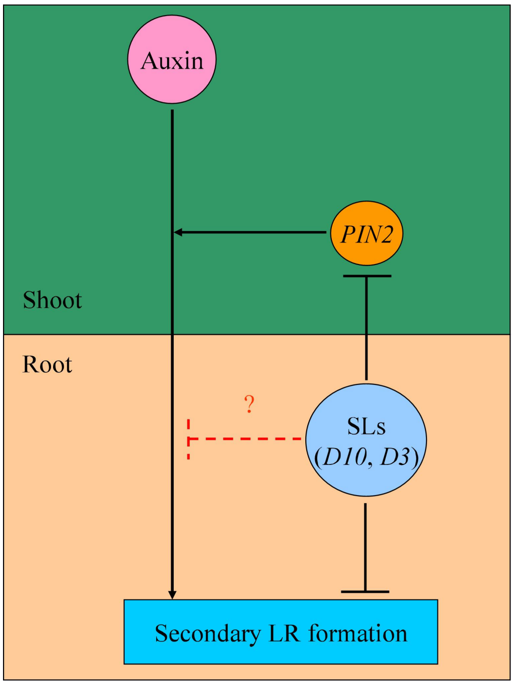

Supplementary FIGURE 3 Summary of the interactions between auxin and strigolactones (SLs)/strigolactones signalling regulate secondary LR formation in rice. The arrow represents promotion. The horizontal line represents inhibition. The dotted red line represents the unknown.
